# Effect of Prolonged-Release Pirfenidone on Renal Function in Septic Acute Kidney Injury Patients: A Double-Blind Placebo-Controlled Clinical Trial

**DOI:** 10.1155/2021/8833278

**Published:** 2021-01-13

**Authors:** Jonathan S. Chávez-Iñiguez, Jorge L. Poo, Miguel Ibarra-Estrada, Leonel García-Benavides, Guillermo Navarro-Blackaller, Cynthia Cervantes-Sánchez, Eduardo Nungaray-Pacheco, Ramón Medina-González, Juan Armendariz-Borunda, Guillermo García-García

**Affiliations:** ^1^Servicio de Nefrología, Hospital Civil de Guadalajara Fray Antonio Alcalde, Guadalajara, Jalisco, Mexico; ^2^Universidad de Guadalajara, Centro Universitario de Ciencias de La Salud CUCS, Guadalajara, Jalisco, Mexico; ^3^Unidad de Investigación, ITESM-CCM, Mexico City, Mexico; ^4^Servicio de Unidad de Terapia Intensiva, Hospital Civil de Guadalajara Fray Antonio Alcalde, Guadalajara, Jalisco, Mexico; ^5^Universidad de Guadalajara, Centro Universitario de Tonalá CUTonalá, Tonalá, Jalisco, Mexico; ^6^Instituto de Biología Molecular en Medicina, CUCS, Universidad de Guadalajara, Guadalajara, Jalisco, Mexico; ^7^Tecnologico de Monterrey, Campus Guadalajara, Monterrey, Mexico

## Abstract

**Background:**

There is no treatment for septic acute kidney injury (sAKI). The anti-inflammatory activity of prolonged-release pirfenidone (PR-PFD) could be beneficial in this clinical setting.

**Methods:**

This study was a double-blind randomized clinical trial in sAKI patients with nephrology consultation at the Civil Hospital of Guadalajara, in addition to the usual treatment of AKI associated with sepsis; patients were randomized to receive either PR-PFD at 1,200 mg/day (group A) or 600 mg/day (group B) or a matched placebo for 7 consecutive days. The primary objective was the decrease in serum creatinine (sCr) and increase in urinary volume (UV); the secondary objectives were changes in serum electrolytes, acid-base status, and mortality.

**Results:**

Between August 2016 and August 2017, 88 patients were randomized. The mean age was 54 (17 ± SD) years, and 47% were male. The main site of infection was the lung (39.8%), septic shock was present in 39.1% of the cases, and the mean SOFA score was 8.8 points. 28 patients received PFD 1,200 mg, 30 patients received PFD 600 mg, and 30 patients received placebo. During the study, sCr did not differ among the groups. The reversion rate of sCr, UV, and mortality was not different among the groups (*p*=0.70, *p*=0.47, and *p*=0.38, respectively). Mild adverse events were not different among the groups.

**Conclusion:**

PR-PFD did not improve the clinical course of sAKI and seemed to be safe in terms of adverse events. This trial is registered with NCT02530359.

## 1. Introduction

Acute kidney injury (AKI) is a serious medical complication that is independently associated with poor outcomes [1, 2]. Approximately one out of five nonsevere sepsis patients develop AKI [3], increasing to two-thirds in critically ill patients [4–9]. Approximately 50% of ICU patients with AKI die, and those who survive an AKI episode have an increased risk of progressing to chronic kidney disease (CKD) [10–12]. Currently, there are few pharmacological therapeutic options available to prevent or treat AKI, and management is limited to alleviate secondary hemodynamic and toxic renal insults and to provide supportive measures, such as dialysis.

Although AKI can be caused by a variety of factors [10], sepsis is the most important etiology [13]. Sepsis-associated AKI (sAKI) is distinct from nonsepsis AKI in terms of pathogenesis, patient characteristics, and clinical outcomes [13–15]. sAKI is more frequent, more severe, less likely to resolve once AKIN stage 3 develops, and associated with higher mortality [16]. It has been suggested that glomerular ultrafiltration of toxic blood is the inciting mechanism for tubular stress and subsequent tubular damage [17]. According to this hypothesis, during sepsis, the glomerular filtrate is loaded with cytokines, chemokines, and complement fragments, which may have a toxic effect on tubular cells [18]. This “inflammatory” hypothesis of AKI is supported by experimental observations [19]. Renal responses to inflammation may be directed to decrease energy consumption by autophagy, digestion, mitochondrial dysfunction (mitophagy), and loss of cell polarity [20]. How these complex inflammatory events affect renal function remains unknown. Therefore, there is a need for additional interventions that could improve AKI prognosis [21]. Experimental studies support anti-inflammatory and positive effects induced by PFD in different AKI models [22–26]. In this study, we examined the effects of PR-PFD on renal function in sAKI patients in a double-blind, randomized, clinical trial.

## 2. Materials and Methods

This study was a randomized, double-blind, parallel-design clinical trial conducted in a single university hospital. The population of the study consisted of hospitalized septic AKI patients. AKI was diagnosed by the serum creatinine (sCr) KDIGO criteria [27]; sepsis was defined according to the Surviving Sepsis-3 campaign [28]. Septic patients with AKI aged ≥ 18 and < 85 years, having baseline creatinine <2 mg/dL, and with written informed consent were included; patients with CKD (basal serum creatinine > 2 mg/dl) stage 3b, 4, or 5, on chronic dialysis, with a history of AKI, or with renal replacement therapy within the last 3 months and pregnant individuals were excluded. In addition to standard AKI treatment, patients were randomly assigned to 3 study groups: group A, oral 1200 mg of PR-PFD every 12 hours for 7 consecutive days; group B, 600 mg of PR-PFD in the morning and a matched placebo at night for 7 consecutive days; and group C, a matched placebo orally every 12 hours for 7 consecutive days. The primary objective was the decrease in serum creatinine (sCr) and increase in urinary volume (UV); the secondary objectives were changes in serum electrolytes, acid-base status, and mortality. Sample size was not determined because this intervention was never tried before. The randomization process was carried out in Excel software in a 1 : 1 : 1 fashion, and nephrology staff members blinded to the allocation groups administered the drugs to each patient every day. All patients had a detailed clinical history and physical examination that included the measurement of blood pressure, heart and respiratory rates, oxygen saturation, ventilatory parameters in patients with mechanically assisted ventilation, and strict fluid balance. Complete blood count, serum creatinine (sCr), serum urea, BUN, serum electrolytes, and urinalysis parameters were measured on a daily basis. The study was approved by the Hospital Civil de Guadalajara Fray Antonio Alcalde Ethics Committee (HCG/CI-0049/15) and by Comision Federal para la Proteccion de Riesgos Sanitarios (COFEPRIS), México, both in June 2016. Written informed consent was obtained from all study participants. The trial was presented according to the CONSORT 2010 Explanation and Elaboration guide.

### 2.1. Statistical Analysis

Continuous variables are presented as the mean (±SD) when normally distributed or as medians (interquartile range (IQR)) in the case of abnormal distribution, following the Shapiro–Wilk test. One-way ANOVA was used to compare differences between groups. Categorical variables are expressed as proportions and were compared with Fisher's chi-square or exact test as appropriate. For the rest of the tests, the *p* values were two-tailed; a *p* < 0.05 was considered statistically significant. Statistical analysis and graphs were produced with MedCalc statistical software version 19.1.3 (Ostend, Belgium).

## 3. Results

**Figure 1:**
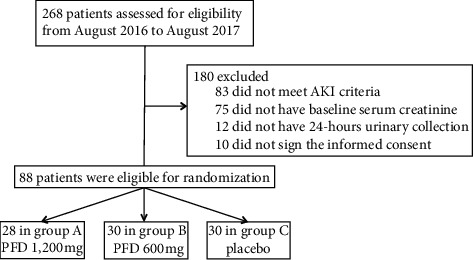
Flowchart of the study patients.

Between August 2016 and August 2017, a total of 268 patients were assessed for eligibility; one hundred eighty patients did not meet the inclusion criteria, and 88 were randomized: 28 in group A (PFD, 1,200 mg), 30 in group B (PFD, 600 mg), and 30 in group C (placebo) (Figure 1). The baseline characteristics of the study patients are shown in Table 1; the mean age was 54 (±SD) years, and 47% were male. The most common infection site was the lung (39.8%), and septic shock was present in 39.1% of the cases. Nonsurgical cases were frequent in our study population (60.2%). Mechanical ventilation was used in 29.5% of the patients, and the mean SOFA score was 8.8 points. The time elapsing from hospitalization to randomization was 6.9 hours (Table 1).

**Figure 2:**
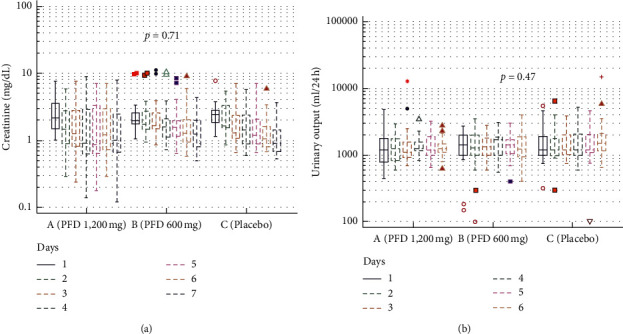
AKI trajectory during the intervention period by sCr (a) and UV (b) according to randomization groups.

The AKI trajectory by sCr during the intervention is presented in Figure 2(a). A tendency toward improvement in sCr was observed in all groups, but it was not significantly different (*p*=0.71) throughout the treatment period. However, in the intragroup analysis, sCr decreased significantly in group B (3.0 mg/dL to 1.7 mg/dL, *p*=0.01) and in group C (2.6 mg/dL to 1.2 mg/dL, *p*=0.01) but not in group A (2.9 mg/dL to 1.8 mg/dL, *p*=0.26). The sCr reversion rate, defined as a decrease in sCr to ≤30% from baseline at any time during hospitalization, was similar in all groups (60.7%, 50%, and 53.3% in groups A, B, and C, respectively, *p*=0.70). The AKI course by urine volume during the intervention is presented in Figure 2(b). UV decreased in all groups, but the change was not statistically significant (from 1,478 ml/day to 1,362 ml/day (*p*=0.55) in group A; from 1,455 ml/day from 1,420 ml/day (*p*=0.98) in group B; and from 1,732 ml/day to 2,364 ml/day (*p*=0.28) in group C). Intermittent hemodialysis (IHD) was required in 14 (16%) patients. In group A, two (7.1%) patients needed IHD; in group B, 6 (20%) patients needed IHD; and in group C, 6 (20%) patients needed IHD. However, the differences were not statistically significant (*p*=0.30). The acid-base status, electrolytes, and other variables during the study period are shown in Table 2. Significant changes in serum pH, serum potassium, and blood hemoglobin were observed only in group B.

Adherence was defined as the intake of the drug/placebo tablets for >80% of the intakes (in total 14 during the 7 days) which was similar among all groups (*p*=0.71). In 82%, 93%, and 80% of the cases, they met the adherence criteria in groups A, B, and C, respectively. No patient required study drug discontinuation during the study.

**Figure 3:**
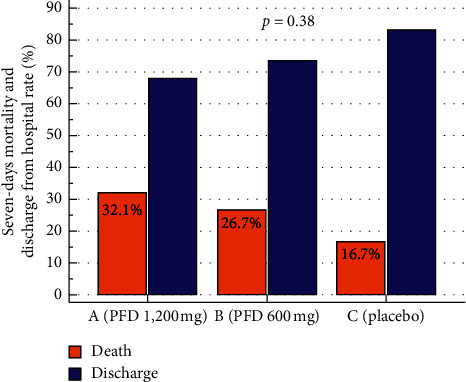
Mortality and discharge from hospital rate according to study groups.

Adverse events were similar among the groups: 42.9% in group A, 43.3% in group B, and 30% in group C (*p*=0.48), the most common being gastrointestinal discomfort (18%), hyperuricemia (9%), and rash (7%). Twenty-two (25%) patients died during the study, 9 (32.1%) patients in group A, 8 (26.7%) patients in group B, and 5 (16.7%) patients in group C (Figure 3), and the majority (77%) died within the first 7 days (*n* = 17) of follow-up. A total of 10 (45.4%) patients died in the ICU. The mortality rate was not different among the groups (*p*=0.38). Patients who received PFD at any dose had a nonsignificant (*p*=0.21) risk ratio for death of 1.1 (95% CI 0.93–1.48) compared to patients in placebo.

## 4. Discussion

In this single-center, double-blind, randomized control trial conducted in septic AKI patients, PR-PFD at two different dosages did not improve kidney function compared to that with the placebo. However, a significant intragroup improvement was observed in group B (PR-PFD, 600 mg/day), as evidenced by a significant decrease in sCr by day 7 of the study, and plausible improvement event also observed in the placebo group.

In sepsis, there are extensive proinflammatory and anti-inflammatory factors that change rapidly once sepsis develops [29]. Previous studies support an anti-inflammatory effect induced by PFD. In the nephrectomized rat model, PFD was effective in decreasing TNF-*α* and IL-6 levels, significantly decreasing proteinuria and NAG activity, attenuating interstitial fibrosis, and decreasing the expression of fibrotic markers and macrophage infiltration. PFD treatment significantly inhibited the expression of TNF-*α*, IL-6, and nitric oxide synthase-2 by M1 macrophages, suggesting its efficacy in the early and late periods of kidney damage [22]. The discrepancy between the study by Chen et al. and our findings regarding TNF-*α* levels could be explained by the etiology of AKI. We explored this event in human sepsis, which is considered a more robust inflammatory response than that in the nephrectomized model; another explanation could be the drug dosing and schedule of administration. To the best of our knowledge, this study is the first time that PR-PFD has been tested in sAKI, and unfortunately, we are not able to compare our findings with other clinical studies.

The primary action of PFD is the blockade of TGF-*β*. It seems reasonable to target TGF-*β* during acute sepsis because the anti-inflammatory and immunoregulatory effects of TGF-*β* involve activation of Smad7, and inhibition of renal inflammation is associated with marked upregulation of renal Smad7 and suppression of NF-*κ*B activation to switch off the inflammatory response [23, 24].

As reported, PFD improved kidney function in a model of acute and chronic kidney disease in rats; PFD prevented the elevation of sCr and BUN in a remnant kidney model of chronic renal failure; histological findings revealed a decrease in interstitial fibrosis. These effects were mediated by the suppression of TGF-*β* and fibronectin mRNA expression [23]. Shimizu et al. showed attenuation of renal damage in a rat model with unilateral obstruction during treatment with PFD and induced renal function recovery before removal of ureteral obstruction [24], and the same group also showed that PFD prevents collagen accumulation in the remanent kidney in rats with partial nephrectomy [25]. However, septic AKI could have another physiopathological mechanism and may differ in the response to the same drug.

Apoptosis has been shown to play a role in the pathogenesis of sepsis nephrotoxicity, and PFD was shown to ameliorate fibrosis [30]. Shihab et al. evaluated the effect of PFD on the expression of apoptosis-regulatory genes in the kidneys of CsA-treated rats. PFD significantly ameliorated nephrotoxicity, and the number of apoptosis-positive cells was reduced by PFD treatment. In addition, PFD downregulated the mRNA expression of CsA-induced p53 and Fas-ligand and increased that of Bcl-xL, which was previously reduced by CsA. Finally, PFD significantly downregulated caspase 3 expression, presented mostly on renal tubular epithelial cells. The authors concluded that PFD could have an antiapoptotic effect [26].

Perhaps PR-PFD (or the dose we implemented) was not sufficient to improve outcomes in sAKI even if there was a plausible effect and maybe the negative effect of PFD 600 mg in our study may be because it did not reach a clinical target dose concentration. Previous trials have demonstrated that, in septic AKI patients, there is a lack of evidence for the improvement of outcomes with well-established therapies. For instance, early goal-directed therapy is not beneficial to renal function, i.e., aggressive fluid loading with a positive fluid balance is not beneficial to renal function and may even be injurious, and artificial colloids could be harmful to the kidney [17].

We realize that our study has the following limitations: this was a single-center study, the sample population was small, and we did not take into account the urinary output to classify AKI; the statistical analysis may have been underpowered for the primary and secondary outcomes; and finally, follow-up was limited to 7 days. Due to these limitations, we know that these results cannot be generalized to other populations.

On the other hand, this study is the first time that PR-PFD has been used to explore the potential improvement in septic AKI patients. Despite our negative results, we believe that PFD should be explored in future studies, and there is evidence of improvement in inflammation and fibrosis processes, which are fundamental in the pathogenesis of AKI [22–26].

## 5. Conclusion

In conclusion, in septic AKI patients, PR-PFD for 7 days did not improve kidney function when compared with placebo, and it was safe in terms of adverse events. Further studies are needed to confirm our results.

## Figures and Tables

**Table 1:** Demographic and clinical characteristics of patients.

	Group A, *n* = 28	Group B, *n* = 30	Group C, *n* = 30	*p*
Male (%)	15 (53.6)	14 (46.7)	13 (43.3)	0.73
Age (years)	55 ± 19.6	54 ± 15.7	53 ± 17.2	0.85
Sepsis site (%)				0.64
Pulmonary	9 (32.1)	12 (40)	14 (46.7)	
Gastrointestinal	6 (21.4)	3 (10)	4 (13.3)	
Urinary	5 (17.9)	5 (16.7)	4 (13.3)	
Soft tissue	7 (25)	7 (23.3)	7 (23.3)	
Central nervous system	1 (3.6)	0	0	
Others	0	3 (10)	1 (3.3)	
Comorbidities (%)				0.71
Neoplasia	1 (3.6)	4 (13.3)	4 (13.3)	
HIV	3 (10.7)	3 (10)	2 (6.7)	
COPD	0	0	1 (3.3)	
CHF	1 (3.6)	2 (6.7)	3 (10)	
DM (%)	10 (35.7)	16 (53.3)	7 (23.3)	0.05
Hypertension (%)	8 (28.6)	16 (53.3)	12 (40)	0.15
Baseline sCr (mg/dL)	0.81 ± 0.33	0.87 ± 0.29	0.78 ± 0.24	0.48
sCr on day 1 (mg/dL)	3.0 ± 1.7	3.3 ± 3.1	2.7 ± 1.6	0.60
Oligoanuria (%)	3 (10.7)	7 (23.3)	6 (20)	0.43
Septic shock (%)	11 (39.3)	10 (34.5)	13 (43.3)	0.78
Cardiogenic shock (%)	4 (14.3)	7 (23.3)	3 (10)	0.35
Surgical case (%)	13 (46.4)	14 (46.7)	8 (26.7)	0.19
Mechanical ventilation (%)	10 (35.7)	6 (20)	10 (33.3)	0.36
Randomization time (h)	6.6 ± 7	5.5 ± 6.7	8.5 ± 11.7	0.40
SOFA score	9 ± 2.3	8.7 ± 2.5	8.9 ± 2.5	0.84

HIV: human immunodeficiency virus; COPD: chronic obstructive pulmonary disease; CHF: chronic heart failure; DM2: diabetes mellitus.

**Table 2:** Variables of the study from day 1 compared to day 7 according to the study group.

	Group A, *n* = 28	Group B, *n* = 30	Group C, *n* = 30	*p*
Serum pH day 1	7.38 ± 0.08	7.35 ± 0.07	7.32 ± 0.1	0.10
Serum pH day 7	7.39 ± 0.06	7.38 ± 0.05	7.40 ± 0.07	0.67
*p* value	1.0	0.01	0.23	
Serum HCO^3−^ (mEq/L) day 1	21.1 ± 5.4	21.3 ± 5.0	21 ± 4.5	0.96
Serum HCO^3−^ (mEq/L) day 7	23.2 ± 4.5	21.9 ± 4.1	24.8 ± 4.8	0.20
*p* value	0.18	0.30	0.08	
Serum K (mEq/L) day 1	4.1 ± 0.9	4.4 ± 1.2	4.3 ± 1.5	0.79
Serum K (mEq/L) day 7	3.6 ± 0.6	3.6 ± 0.6	3.7 ± 0.6	0.87
*p* value	0.14	0.01	0.13	
Serum Na (mEq/L) day 1	137 ± 7.2	135 ± 9.1	131 ± 25.9	0.39
Serum Na (mEq/L) day 7	133 ± 5.5	135 ± 7.1	138 ± 5.7	0.07
*p* value	0.11	0.89	0.42	
Serum Ca (mEq/L) day 1	9.7 ± 7.2	7.6 ± 9.1	7.9 ± 25.9	0.40
Serum Ca (mEq/L) day 7	7.6 ± 0.7	7.6 ± 1.0	7.7 ± 1.3	0.98
*p* value	0.33	0.94	0.43	
Serum PO4 (mg/dL) day 1	4.0 ± 1.4	4.4 ± 1.9	4.2 ± 1.4	0.67
Serum PO4 (mg/dL) day 7	3.6 ± 1.4	3.6 ± 1.6	3.6 ± 1.4	0.99
*p* value	0.85	0.11	0.17	
Blood Hgb (g/dL) day 1	10.1 ± 1.9	10.2 ± 2.1	10.2 ± 2.2	0.98
Blood Hgb (g/dL) day 7	9.7 ± 2.3	9.3 ± 2.3	10.2 ± 2.7	0.45
*p* value	0.14	0.01	0.64	
Serum uric acid (mg/dL) day 1	4.7 ± 2.3	5.7 ± 3.2	6.2 ± 4.6	0.48
Serum uric acid (mg/dL) day 7	3.7 ± 1.9	6.1 ± 3.3	6.2 ± 5.9	0.14
*p* value	0.52	0.75	0.45	
Leukocytes day 1 (10^3^)	13.1 ± 6.4	14.8 ± 6.0	11.8 ± 5.3	0.16
Leukocytes day 7 (10^3^)	10.7 ± 5.8	12.8 ± 6.5	9.9 ± 4.8	0.21
*p* value	0.51	0.14	0.19	
Platelets day 1 (10^3^)	206 ± 149	265 ± 182	279 ± 169	0.22
Platelets day 7 (10^3^)	295 ± 164	266 ± 144	298 ± 147	0.71
*p* value	0.10	0.72	0.06	

## Data Availability

The data used to support this trial can be found at the Nephrology service of the Civil Hospital of Guadalajara Fray Antonio Alcalde.
